# CCX559 is a potent, orally-administered small molecule PD-L1 inhibitor that induces anti-tumor immunity

**DOI:** 10.1371/journal.pone.0286724

**Published:** 2023-06-07

**Authors:** Kathleen M. C. Sullivan, Marta Vilalta, Linda S. Ertl, Yu Wang, Carolyn Dunlap, Karen Ebsworth, Bin N. Zhao, Shijie Li, Yibin Zeng, Zhenhua Miao, Pingchen Fan, Venkat Mali, Christopher Lange, Darren McMurtrie, Ju Yang, Rebecca Lui, Ryan Scamp, Vicky Chhina, Alice Kumamoto, Simon Yau, Ton Dang, Ashton Easterday, Shirley Liu, Shichang Miao, Israel Charo, Thomas J. Schall, Penglie Zhang

**Affiliations:** ChemoCentryx, Inc., San Carlos, California, United States of America; Kansai Medical University: Kansai Ika Daigaku, Institute of Biomedical Science, JAPAN

## Abstract

The interaction of PD-L1 with PD-1 is a major immune checkpoint that limits effector T cell function against cancer cells; monoclonal antibodies that block this pathway have been approved in multiple tumor indications. As a next generation therapy, small molecule inhibitors of PD-L1 have inherent drug properties that may be advantageous for certain patient populations compared to antibody therapies. In this report we present the pharmacology of the orally-available, small molecule PD-L1 inhibitor CCX559 for cancer immunotherapy. CCX559 potently and selectively inhibited PD-L1 binding to PD-1 and CD80 *in vitro*, and increased activation of primary human T cells in a T cell receptor-dependent fashion. Oral administration of CCX559 demonstrated anti-tumor activity similar to an anti-human PD-L1 antibody in two murine tumor models. Treatment of cells with CCX559 induced PD-L1 dimer formation and internalization, which prevented interaction with PD-1. Cell surface PD-L1 expression recovered in MC38 tumors upon CCX559 clearance post dosing. In a cynomolgus monkey pharmacodynamic study, CCX559 increased plasma levels of soluble PD-L1. These results support the clinical development of CCX559 for solid tumors; CCX559 is currently in a Phase 1, first in patient, multicenter, open-label, dose-escalation study (ACTRN12621001342808).

## Introduction

Cancer cells escape tumor-specific T cell responses via engagement of the inhibitory immune receptor PD-1, which negatively regulates effector responses essential for elimination of cancers [1, 2]. Activation of PD-1 by PD-L1 inhibits T cell receptor downstream signaling via SHP2-mediated dephosphorylation; a key target of PD-1 is the costimulatory receptor CD28 [3]. Several monoclonal antibody therapeutics that target PD-1 or PD-L1 by blocking their interaction have been approved in multiple tumor indications [4]. PD-L1 also binds CD80, a ligand for CD28, *in cis* on the same cell surface [5]: this interaction may reduce CD28 signaling by competing for CD80 [6], or by co-localizing activated PD-1 with CD28 on the T cell surface [3].

Small molecule PD-L1 inhibitors have distinct properties that may be advantageous when compared to approved monoclonal antibody therapies [7–9]. Small molecule drugs typically exhibit a higher rate of clearance compared to antibodies, and the ability to rapidly eliminate drug may reduce the magnitude of immune-related adverse events, as well as the need for corticosteroid treatment. Such compounds are expected to elicit minimal immunogenicity, and thus minimize adverse events induced by anti-drug antibodies. Therapeutic efficacy of small molecule inhibitors may be enhanced by increased tumor exposure compared to normal tissues via diffusion-based distribution [10], particularly for bulky tumors or tumors with high interstitial fluid pressure. In contrast, antibody distribution occurs via the fluid phase, which may lead to higher relative accumulation in tissues and to increased risk of adverse events.

In this report we describe the *in vitro* and *in vivo* pharmacology of the small molecule CCX559. We provide evidence supporting CCX559 as a selective, potent inhibitor of PD-L1, its mechanism of action, and ability to enhance primary human T cell activation. Studies were performed *in vivo* to measure anti-tumor activity and drug properties; orally-dosed CCX559 had significant anti-tumor efficacy in two murine models, accumulated in the tumors, and induced the reversible internalization of PD-L1. Where appropriate, anti-PD-L1 monoclonal antibody was included as a comparator, and CCX559 showed equivalent PD-L1 inhibition. Our results support the continued clinical development of CCX559 as a PD-L1 inhibitor in oncology.

## Results

### CCX559 potently and selectively inhibited human PD-L1 *in vitro*

**Fig 1:**
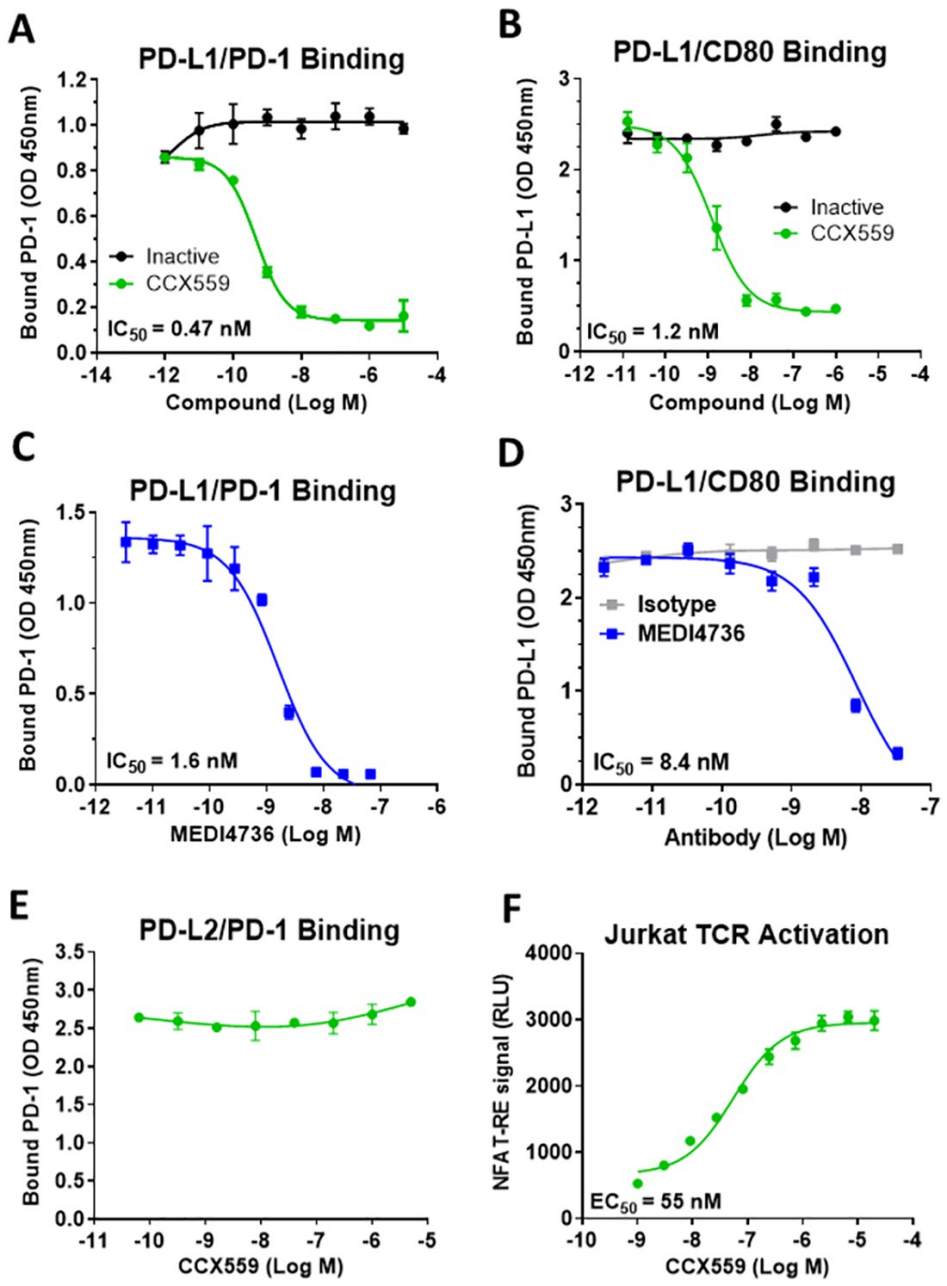
CCX559 selectively inhibited *in vitro* binding of human PD-L1 to PD-1 and CD80, and enhanced TCR signaling in Jurkat cells. (A) Recombinant PD-1 binding to plate-bound PD-L1 was inhibited by CCX559 with an IC_50_ of 0.47 nM, as shown in a representative assay (green circles). An inactive compound with a similar structure to CCX559 had no effect on binding (black circles). (B) PD-L1 binding to plate-bound CD80 was inhibited by CCX559 (green circles) but not by the inactive compound (black circles). (C) The anti-PD-L1 antibody MEDI4736 inhibited PD-L1 binding to PD-1 in the same assay as in A. (D) CD80 binding was also inhibited by MEDI4736 (blue squares), but not by the isotype control (grey squares) in the same assay as B. (E) PD-1 binding to PD-L2 was not affected by up to 5 μM CCX559. (F) CCX559 enhanced TCR activation in a Jurkat cell-based NFAT reporter assay performed in 100% FBS to mimic physiological conditions. The graph is representative of all assays performed. EC_50_ and IC_50_ values were calculated with GraphPad Prism using 3-parameter nonlinear regression. The error bars represent ± one standard deviation (SD, n = 3 replicates).

CCX559 inhibited the binding of recombinant human PD-L1 to PD-1 (Fig 1A) and to CD80 (Fig 1B) with low- or sub-nanomolar potency in plate-based binding assays. An inactive compound, which is structurally similar to CCX559, did not inhibit PD-L1 binding to PD-1 or CD80 (Fig 1A and 1B). MEDI4736, a research grade anti-hPD-L1 antibody with the same amino acid sequence as the clinically-approved durvalumab [11], had comparable activity to CCX559 in inhibiting PD-L1 interaction with PD-1 and CD80 (Fig 1C and 1D).

Selectivity for PD-L1 was assessed by measuring CCX559 inhibitory activity against PD-L2, which is the most closely related member of the B7 family of immune-regulatory ligands, with 34% identity to PD-L1 in the ectodomain [12]. PD-L2 is also a ligand for PD-1, but does not associate with PD-L1 or CD80. CCX559 had no effect on the interaction between PD-1 and PD-L2 (Fig 1E), which is readily blocked by an anti-PD-1 antibody (S1 Fig). CCX559 was also tested against TIGIT, another immune-regulatory ligand within the larger immunoglobulin superfamily, and did not inhibit the interaction with its receptor CD155 (S1 Fig).

To measure the effect of CCX559 on PD-1-mediated suppression of T cell receptor (TCR) signaling, Jurkat cells expressing PD-1 were activated by CHO cells expressing both a TCR activator and PD-L1. CCX559 dose-dependently increased TCR signaling in the Jurkat cells as measured by an NFAT-driven reporter (Fig 1F). CCX559 had no effect on TCR signaling when Jurkat cells were activated by cells that expressed a TCR activator but not PD-L1 (S1 Fig).

### CCX559 induced human PD-L1 dimerization and internalization

**Fig 2:**
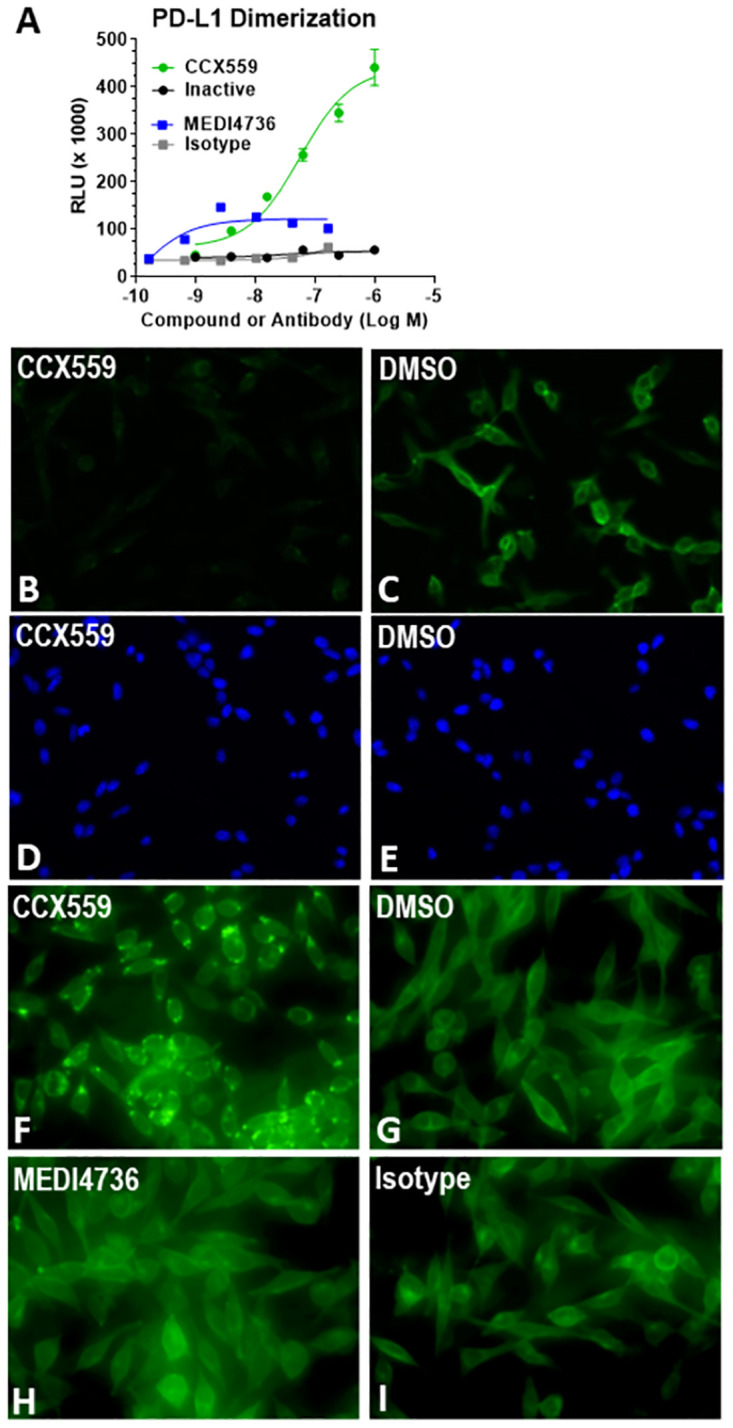
CCX559 induced human PD-L1 dimerization and relocalization to intracellular vesicles *in vitro*. (A) In the PathHunter^®^ PD-L1 Dimerization Assay, CCX559 (green circles) induced a dose-dependent dimerization signal, measured as RLU, but the inactive compound (black circles), MEDI4736 (blue squares) and the isotype control antibody (grey squares) had no or minimal effect. The error bars represent ± SD (n = 3). (B and C) Treatment of MC38-hPD-L1 cells for 5 hours with 1 μM CCX559 (B) reduced surface PD-L1 levels compared to a 0.1% DMSO vehicle control using the same exposure time (C). (D and E) The corresponding DAPI staining shows similar cell densities. (F through H) To detect intracellular PD-L1, MC38-hPD-L1 cells were permeabilized after treating with 300 nM CCX559 (F), 0.1% DMSO (G), 67 nM MEDI4736 (H) or isotype matched control antibody (I) for 22 hours. PD-L1 was observed in intracellular vesicles only in CCX559-treated cells. The images are representative of the entire cell field for each treatment and had identical exposure times.

Small molecule PD-L1 inhibitor compounds with substituted biphenyl-based scaffolds have been reported to bind within a pocket formed by two PD-L1 monomers, resulting in a dimer structure unable to interact with PD-1 [13–15]. As a biphenyl-based inhibitor [16], CCX559 was assayed for dimerization activity in live cells with an engineered U2OS cell system, which expressed two human PD-L1 proteins fused to complementary β-galactosidase fragments. Treatment with CCX559, but not the inactive compound or MEDI4736, dose-dependently induced the dimerization-linked enzymatic signal (Fig 2A).

Small molecule PD-L1 inhibitors have also been demonstrated to induce internalization from the cell surface [14]. The cellular distribution of PD-L1 was thus examined in an MC38 cancer cell line engineered to express human PD-L1: cell surface PD-L1 was visibly reduced in CCX559 treated cultures compared to cells in the control culture within 5 hours (Fig 2B–2E). Cell membrane permeabilization revealed that CCX559, but not the vehicle control, induced PD-L1 redistribution to intracellular vesicles (Fig 2F and 2G). No effect on PD-L1 cell surface levels was observed when cells were cultured with MEDI4736 under identical conditions, nor did MEDI4736 induce PD-L1 localization to intracellular vesicles (Fig 2H and 2I).

### CCX559 increased primary human T cell activity *in vitro*

**Fig 3:**
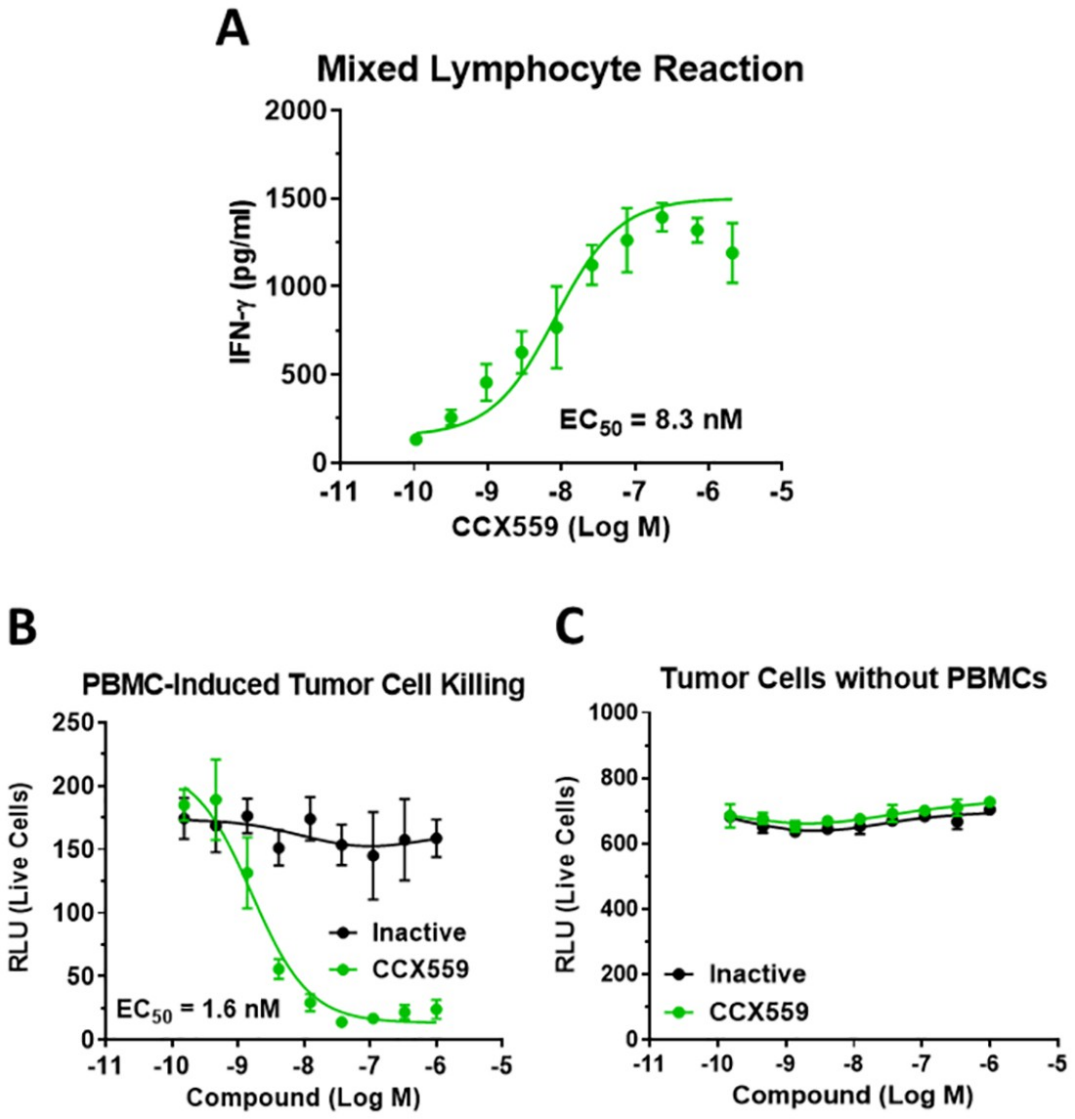
CCX559 enhances the function of primary human T cells *in vitro*. (A) CCX559 increased human T cell IFNγ secretion in a mixed lymphocyte reaction. Total CD4^+^ T cells and monocyte-derived dendritic cells from separate healthy donors were combined in a 1:10 cell ratio, and IFNγ was measured in the culture supernatant after 5 days. The graph shows a representative allogeneic donor pair out of 3 total. (B) A375-eGFP cells and SEB-stimulated human PBMCs were incubated together with CCX559 (green circles), which dose-dependently reduced the number of live cells as measured by eGFP (RLU). The inactive compound (black circles) had no effect on cell killing. (C) In the absence of PBMCs, the same CCX559 doses had no effect on cell survival.

CCX559 was tested for the ability to induce primary human T cell cytokine secretion and cytotoxicity, key T cell responses that are sensitive to PD-1/PD-L1 inhibition. IFNγ secretion was activated in human CD4^+^ T cells by incubating them with allogeneic monocyte-derived dendritic cells (moDC) in a mixed lymphocyte reaction. Treatment with CCX559 dose-dependently increased IFNγ secretion in 3 T cell–moDC donor pairs (Fig 3A). T cell cytotoxicity was measured by combining human PBMCs, which were pre-treated with the superantigen SEB to activate T cells, and A375-eGFP tumor cells that express PD-L1. Tumor cell killing was incomplete as measured by the eGFP signal from live cells; the addition of CCX559, but not the inactive compound, increased hPBMC-mediated killing, resulting in the near complete elimination of eGFP (Fig 3B). CCX559 did not directly affect A375-eGFP cell viability, indicating activity in the assay was hPBMC dependent (Fig 3C).

### CCX559 had potent anti-tumor activity in two murine models *in vivo*

**Fig 4:**
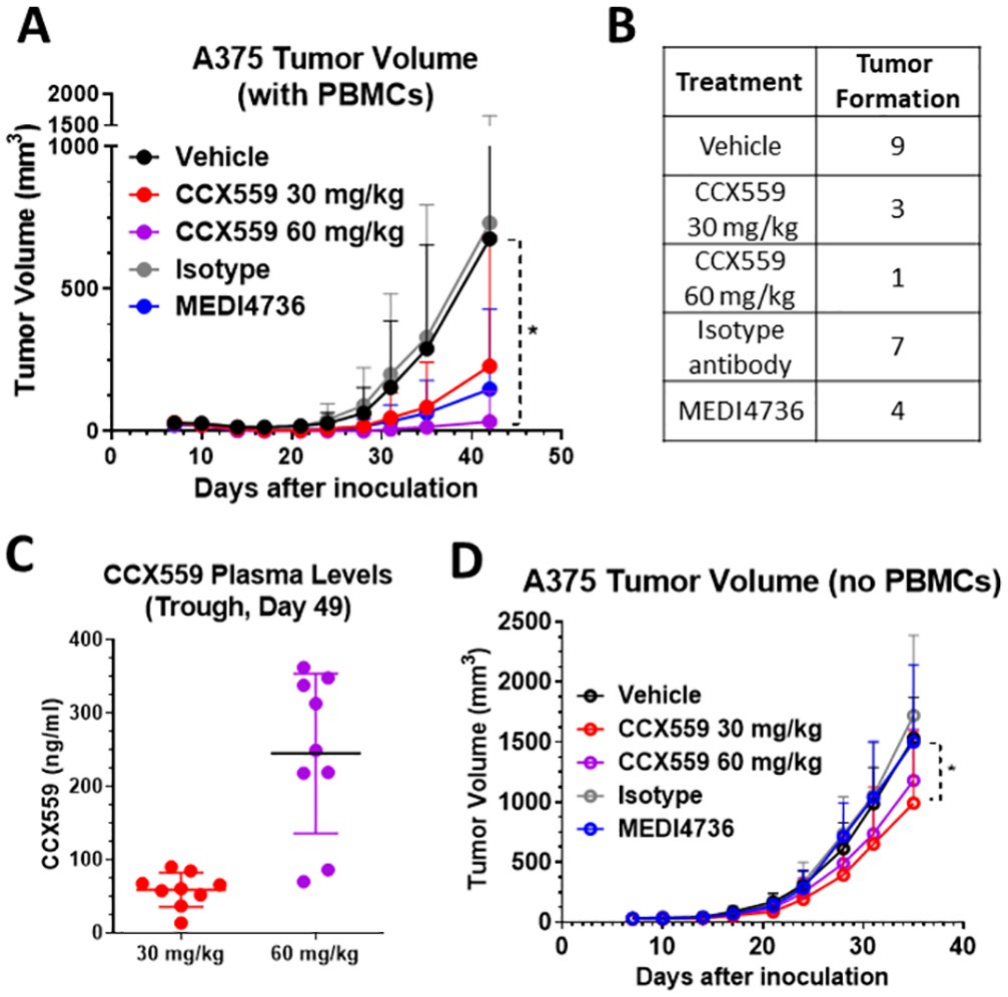
CCX559 reduced tumor formation and growth in the A375 xenograft model with human PBMCs. (A) CCX559, MEDI4736, and control treatments were started on the day after inoculation of NOD.SCID mice with A375 tumor cells and hPBMCs: CCX559 was dosed orally once daily and MEDI4736 was dosed intraperitoneally every 5 days for 5 doses. Only the 60 mg/kg CCX559 group had significantly reduced tumor formation and growth compared to control. (B) The number of mice per group (out of n = 10) that formed A375 tumors was lower in CCX559 and MEDI4736 treated groups compared to vehicle and isotype controls, respectively. (C) CCX559 concentration in blood plasma was measured on day 49, 24 hours after the last dose. (D) In the absence of hPBMCs, all mice in each group (n = 10) successfully formed A375 tumors. No group showed significant reduction of tumor volume at any timepoint, except for 30 mg/kg CCX559 on the last day of study. All data are represented as mean ± SD, *denotes p < 0.05. Student’s t-test was used to compare MEDI4736 vs. isotype-matched antibody and one-way ANOVA with multiple comparisons for CCX559 dose groups vs. vehicle.

The ability of CCX559 to induce human T cell-mediated tumor killing *in vivo* was assessed in the A375 xenograft model, in which human A375 tumor cells and PBMCs were implanted into immune deficient NOD.SCID mice. PD-L1 expressed on the A375 cells reduces anti-tumor activity of the hPBMCs, allowing for tumor formation and growth. At 60 mg/kg CCX559 showed significant anti-tumor activity, preventing tumor formation in all but one mouse out of ten and reducing tumor growth in the remaining mouse compared to the vehicle arm, where all but one of ten animals formed tumors (Fig 4A and 4B). At 30 mg/kg CCX559 prevented tumor formation in seven mice out of ten, but the overall reduction in tumor growth was not significant. Consistent with anti-tumor activity, trough plasma levels of CCX559 were also dose dependent (Fig 4C). MEDI4736 prevented A375 tumor formation in six of ten mice compared to three of ten in the isotype group (blue versus gray circles), with a nonsignificant reduction in tumor growth. As a control, A375 cells were implanted without hPBMCs, resulting in all NOD.SCID mice forming rapidly growing tumors. CCX559 did not significantly inhibit A375 tumor formation or growth in a dose-dependent manner, indicating that the anti-tumor activity is dependent on the presence of human PBMCs (Fig 4D). There is a small reduction in tumor size on the last day of study in the 30 mg/kg CCX559 group only; the mechanism is unclear, but an on-target effect can’t be ruled out, given the possibility that the human PD-L1 on A375 cells could activate PD-1 on murine myeloid and NK cells.

**Fig 5:**
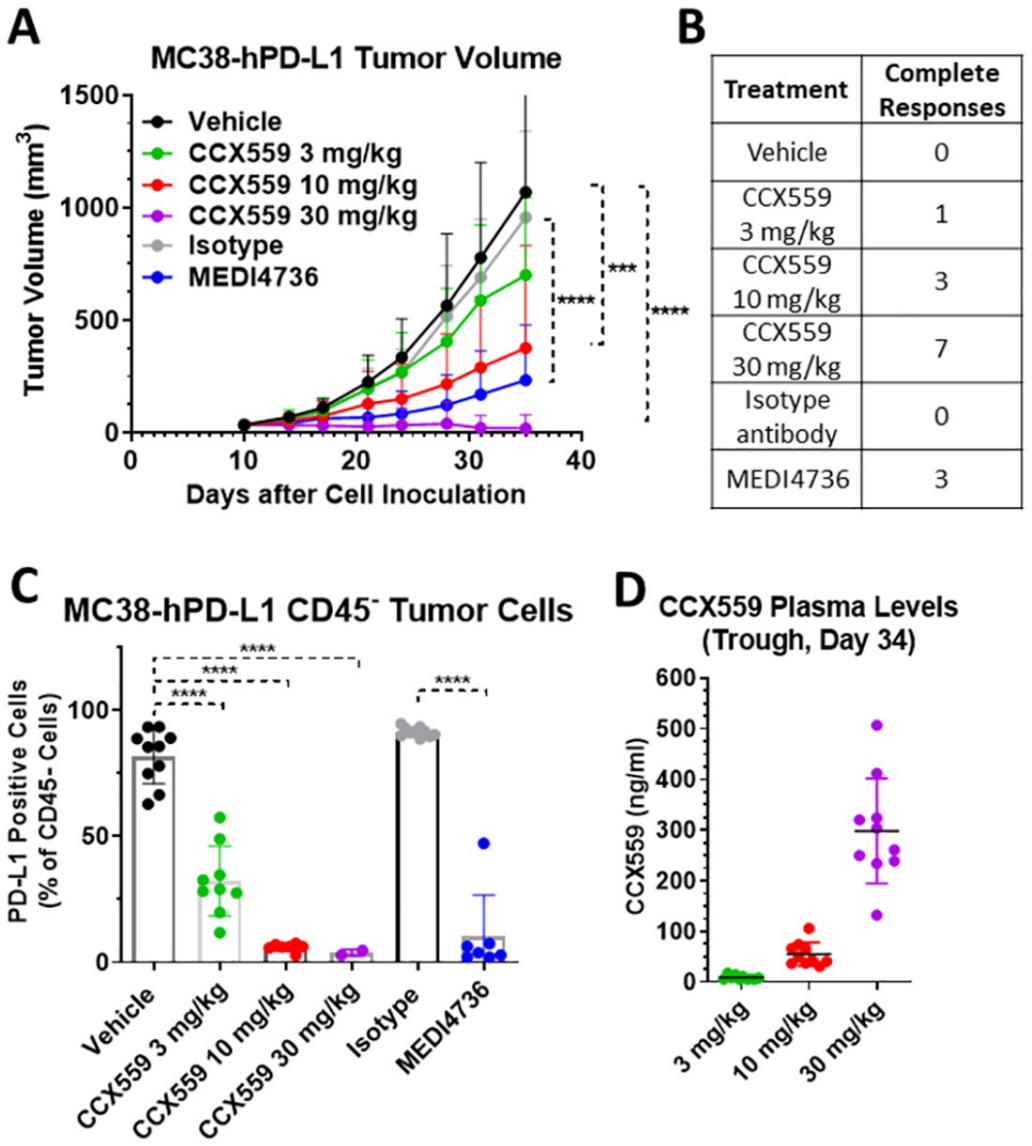
CCX559 dose-dependently suppressed MC38-hPD-L1 tumor growth, induced tumor regression and reduced tumor cell surface PD-L1 levels. CCX559, MEDI4736, and control treatments were started in C57BL/6 mice after tumor formation (>30 mm^3^) on day 11 post MC38-hPD-L1 cell inoculation: CCX559 was dosed orally once daily, and MEDI4736 was dosed intraperitoneally every 5 days for 3 doses. (A) CCX559 (10 and 30 mg/kg) and MEDI4736 significantly reduced average tumor volumes at day 35, compared to their respective controls. (B) The number of mice with a complete response, i.e. no detectable tumor remaining, is shown for each group (n = 10). (C) The percent of CD45- cells in dissociated tumors with detectable cell surface hPD-L1 was reduced by CCX559 and MEDI4736 treatment. (D) Plasma CCX559 concentrations were measured 24 hours after the last dose. All data are represented as mean ± SD, ***denotes p < 0.001, ****denotes p < 0.0001. Statistical analyses for the study arms were the same as Fig 4.

To demonstrate *in vivo* CCX559 anti-tumor activity in mice with an intact immune system, we used the MC38-hPD-L1 model, in which the tumor cells express human and not mouse PD-L1. CCX559 does not cross-react with mouse PD-L1 (S1 Fig), but does inhibit human PD-L1 interaction with mouse PD-1 at a potency similar to human PD-1 (S1 Fig). The MC38-hPD-L1 model has been shown to be responsive to human PD-L1 blockade [17], which is consistent with the reported finding that human PD-L1 activates mouse PD-1 at a potency similar to mouse PD-L1 [12]. CCX559 significantly and dose-dependently reduced tumor growth in the MC38-hPD-L1 model (Fig 5A), when administered orally once per day starting after tumor formation. Complete tumor responses were observed in the CCX559-treated mice, with the majority of tumors (7 of 10) eliminated at the highest dose of CCX559 (30 mg/kg, Fig 5B). MEDI4736 had similar anti-tumor activity, inducing complete responses (3 of 10 mice) and a significant reduction in tumor volume relative to the isotype control (Fig 5A and 5B). Target engagement in tumors was assessed indirectly with an anti-PD-L1 monoclonal antibody, MIH1, that competes with CCX559 and MEDI4736 for binding to human PD-L1 (S2 Fig). MIH1 binding to tumor cells was reduced in a dose-dependent fashion from the vehicle group (80% of CD45- cells positive for MIH1) to 30 mg/kg CCX559 (3.8% of cells positive), and similarly in the MEDI4736 tumors compared to isotype (Fig 5C). CCX559 plasma levels were measured at the end of study (Fig 5D) and after the first 3 days of dosing (S2 Fig), demonstrating that drug exposure was consistent and dose-dependent throughout the treatment period, and that plasma exposures were similar in the significantly efficacious groups across studies (30 mg/kg in MC38-hPD-L1 and 60 mg/kg in A375 xenograft).

**Fig 6:**
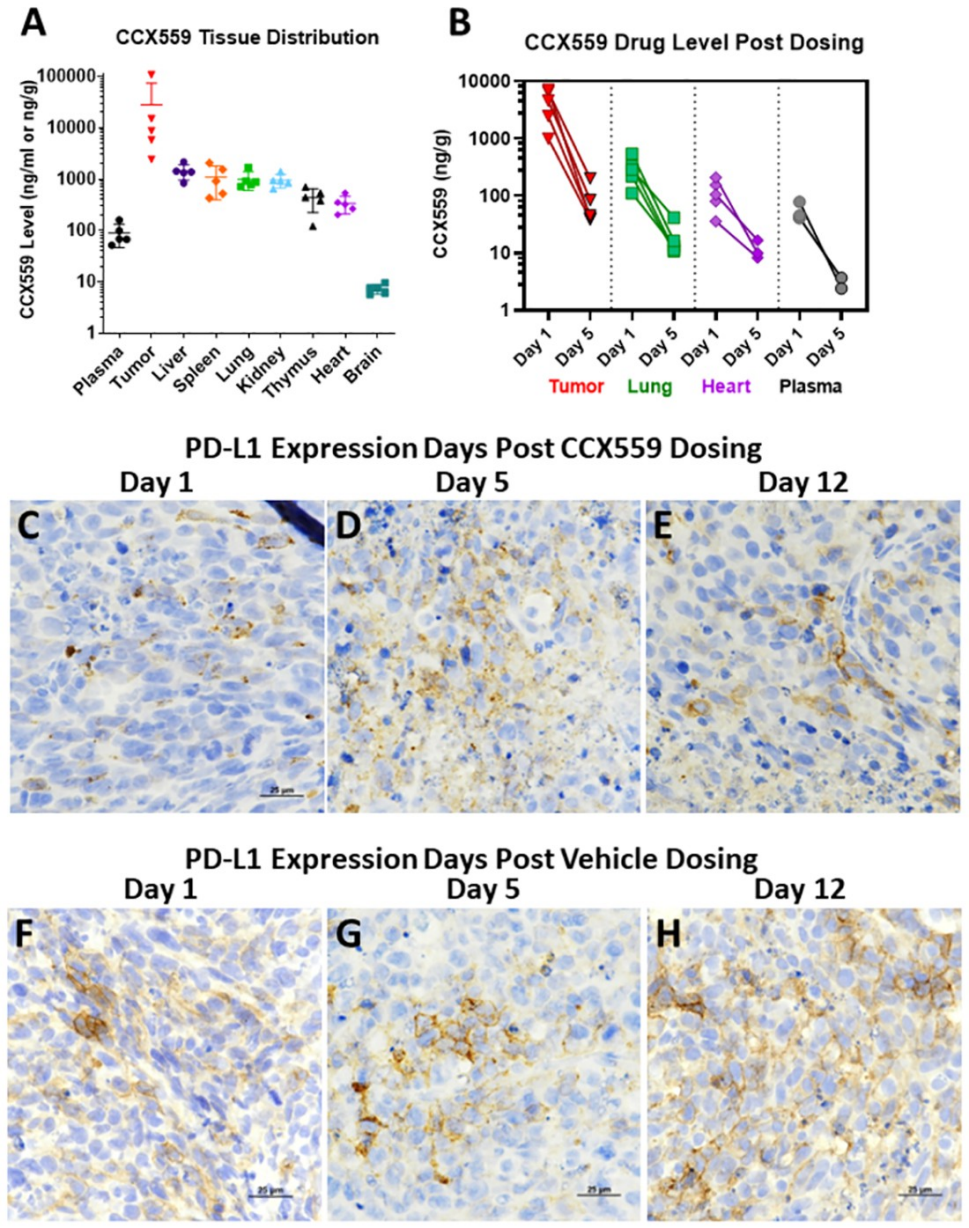
CCX559 accumulated in MC38-hPD-L1 tumors *in vivo* and induced PD-L1 internalization. (A) CCX559 or vehicle was dosed orally once daily for seven days in human PD-L1 KI mice (n = 5 per group) with ≥100 mm^3^ MC38-hPD-L1 tumors. CCX559 levels were measured one day after the final dose and normalized per gram of tissue, or per ml of plasma. (B) In a CCX559 dosing study similar to A, tumor, plasma, and tissue drug levels were measured on days 1, 5 and 12 post dosing. CCX559 levels decreased from day 1 to day 5 and were not detected on day 12. (C-E) For the dosing study in B, only intracellular PD-L1 was observed by IHC with anti-PD-L1 monoclonal antibody 28–8, when CCX559 was still present in tumors on day 1 (C) and day 5 post dose (D), but after drug clearance on day 12 post dose plasma membrane PD-L1 was observed in tumors (E). (F-H) Cell surface PD-L1 was detected in the vehicle control tumors at all timepoints.

To assess *in vivo* CCX559 distribution and clearance, the MC38-hPD-L1 model was repeated in a human PD-L1 knock-in (KI) mouse strain. Oral dosing of 30 mg/kg CCX559 was started at an average tumor size of 100 mm^3^ once per day for 7 days; drug level was measured in plasma, organs, and tumors on day 1 post dosing. The average CCX559 level on day 1 post dose was higher in tumors than plasma and other organs (Fig 6A: 27.9 μg/g tumor vs. 0.007–1.4 μg/g). To confirm this result and assess target engagement and recovery post dosing, the study was repeated with timepoints taken at days 1, 5, and 12 post dosing. Dosing CCX559 for 7 days significantly reduced hPD-L1-MC38 tumor growth compared to vehicle control, and the reduction in tumor volume persisted post dosing until the end of study (S3 Fig). The average CCX559 level on day 1 post dose was higher in tumors than plasma and other organs, but dropped by 98% in all tissues by day 5 post dose (Fig 6B shows plasma, tumors, lung, and heart). No drug was detected by day 12 post dose, suggesting complete clearance. In CCX559-treated tumors hPD-L1 was primarily detected in intracellular vesicles on days 1 and 5 post dose, with some membrane staining observed by day 12 (Fig 6C–6E). In vehicle control tumors PD-L1 was detected at the plasma membrane at all timepoints (Fig 6F–6H).

### Preclinical development of CCX559

**Fig 7:**
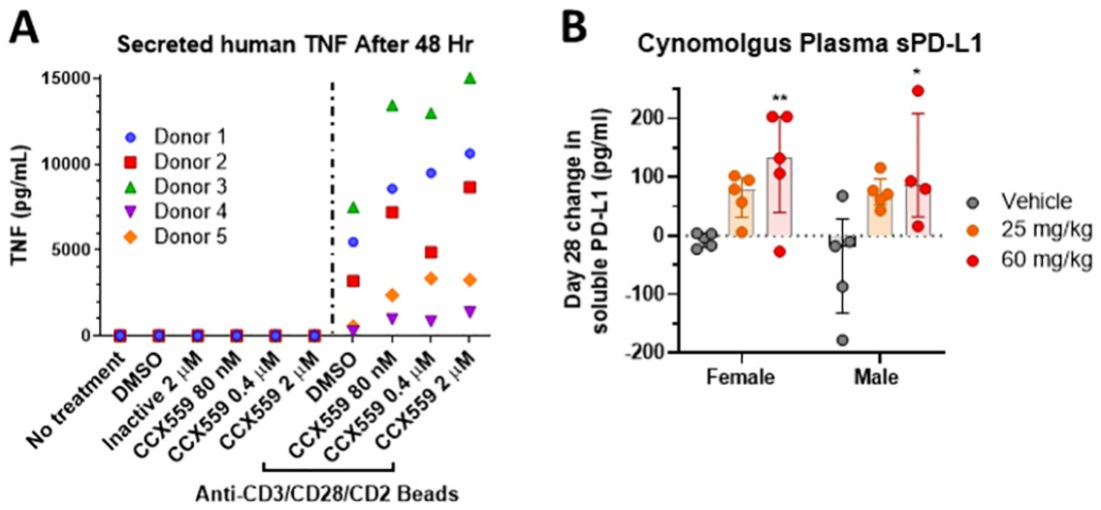
CCX559 did not activate PBMCs in the absence of T cell activation, and CCX559 treatment increased soluble PD-L1 in the plasma of cynomolgus monkeys. (A) TNF secretion from five PBMCs donors was measured after 48 hours of treatment with CCX559 or DMSO, alone or in the presence of anti-CD3/CD28/CD2 microbeads. All donor PBMCs secreted TNF when stimulated by the CD3/CD28/CD2 beads, but CCX559 treatment alone induced no TNF secretion. (B) Soluble PD-L1 levels in cynomolgus monkey plasma were measured after 28 days of treatment with a vehicle control, 25 or 60 mg/kg CCX559. The change in sPD-L1 plasma levels was calculated by subtracting the concentration at predose from day 28 for each animal, and was significantly increased by 60 mg/kg CCX559 in both genders. The average predose plasma sPD-L1 levels ranged from 77 to 148 pg/ml and were not significantly different between groups. One-way ANOVA with Dunnett’s multiple comparisons was used to compare the day 28 change in sPD-L1 between dose groups for each gender. All data are represented as mean ± SD, *denotes p < 0.05, and ** p < 0.01.

Therapeutics that increase immune system activity may cause cytokine release syndrome (CRS), an acute, life-threatening event resulting from systemic pro-inflammatory cytokine release by immune cells [18]. To mitigate this risk in the clinic, CCX559 was evaluated for its ability to induce the *in vitro* release of cytokines from the PBMCs of five healthy human donors. In the absence of T cell activation, CCX559 by itself did not induce release of the pro-inflammatory cytokine TNF when tested at concentrations of up to 2 μM (Fig 7A). CD3/CD2/CD28 beads were used to confirm the PBMCs were capable of releasing cytokines in response to T cell activation. CCX559 increased the secretion of TNF by CD3/CD2/CD28-stimulated PBMCs, consistent with PD-L1/PD-1 blockade downstream of TCR activation. The secretion of other cytokines implicated in CRS, IL-6 and IFNγ, was also not induced by CCX559 alone (S4 Fig).

CCX559 pharmacodynamics were examined in cynomolgus monkeys, because CCX559 exhibited cross species potency *in vitro*, inhibiting the cynomolgus PD-L1/PD-1 interaction (IC_50_ = 0.21 nM) and enhancing cynomolgus T cell activation (S5 Fig). Day 1 and day 28 samples from a GLP toxicology study in non-tumor bearing cynomolgus monkeys provided an opportunity to identify potential clinical biomarkers, even though the study was not designed to detect pharmacodynamic activity. Soluble PD-L1 levels on day 28 were increased by CCX559 treatment compared to baseline levels on day 1. The increase in plasma sPD-L1 on day 28 was significant in the 60 mg/kg dose groups for both genders compared to vehicle control (Fig 7B). Increased in IL-6 levels were also observed in animals from the 60 mg/kg groups compared to vehicle controls (S5 Fig).

## Discussion

The studies described herein demonstrate that the small molecule compound CCX559 is a highly potent and selective inhibitor of human PD-L1. CCX559 prevented human PD-L1 interaction with PD-1 and CD80 at low or sub-nanomolar potency *in vitro*, but had no effect on the interaction of PD-1 with PD-L2, the B7 family member most similar to PD-L1. CCX559 significantly and robustly enhanced primary human T cell responses *in vitro*, measured by IFNγ secretion and tumor cell killing. As expected for a PD-L1 inhibitor, the enhancement of T cell responses by CCX559 was demonstrated to be dependent on activation of the TCR, both in a cell-based TCR reporter assay and a cytokine release assay with hPBMCs.

Despite the lack of cross-reactivity with mouse PD-L1, CCX559 demonstrated dose-dependent anti-tumor activity in two mouse models: the MC38-hPD-L1 model, where human PD-L1 on the tumor cells activates PD-1 to suppress anti-tumor responses of the mouse immune system; and the xenograft model, where human PD-L1 on A375 tumors activates PD-1 on hPBMCs to inhibit cytotoxic immune responses. We demonstrated CCX559 engagement with hPD-L1 *in vivo* through loss of MC38-hPD-L1 tumor cell surface expression by flow cytometry and IHC, and the appearance of intracellular PD-L1 detected by IHC. PD-L1 localization correlated well with tumor levels of CCX559: at 1 and 5 days post dosing when CCX559 is present in tumors, PD-L1 is intracellular; at 12 days when CCX559 has been cleared from tumors, cell surface PD-L1 is detected again. The accumulation of CCX559 in tumors compared to plasma and other organs suggests that CCX559 is readily and preferentially distributed within the tumor microenvironment in this model. The rapid clearance of CCX559 in all tissues tested is consistent with the short half-life measured in mice and within typical range for small molecule drugs. These results suggest that a checkpoint inhibitor with the distribution and clearance properties of CCX559 could be dosed to effectively stimulate anti-tumor responses while limiting immune-related adverse events in the periphery.

The activity of CCX559 was similar to MEDI4736 (durvalumab) *in vitro* and *in vivo*, and consistent with PD-L1 blockade; however, the mechanisms of action are distinct. Published studies show durvalumab, similar to other PD-L1 monoclonal antibody therapeutics, directly competes with PD-1 by binding to an epitope in the IgV domain of PD-L1 that overlaps with the PD-1 binding site [19, 20]. Instead of directly competing with PD-1, CCX559 induces PD-L1 dimerization, which prevents the interaction with PD-1 by occluding the PD-1 binding site. The rapid internalization of cell surface PD-L1 induced by CCX559 provides a second mechanism by which CCX559 prevents interaction with PD-1. Because CCX559 inhibits the CD80/PD-L1 interaction, PD-L1 internalization should not affect the cell surface level of CD80 or CD28 co-stimulation during T cell activation. In contrast, MEDI4736 has no effect on PD-L1 dimerization or cell surface levels in our assays, consistent with published reports [21].

To support the clinical development of CCX559, pharmacodynamic biomarkers were explored opportunistically in cynomolgus monkeys. Treatment with CCX559 increased the level of plasma sPD-L1, which reflects both exosomal PD-L1 and the extracellular domain that is shed from the cell surface by matrix metalloproteases. Increased blood levels of sPD-L1 have been observed in cancer, autoimmunity, inflammation, and pregnancy [1, 22], possibly as a consequence of PD-L1’s role in maintaining peripheral homeostasis [1, 23]. A number of cytokines, including IFNγ, have been shown to increase sPD-L1 levels *in vitro* and *in vivo* [24, 25]. The elevation of plasma sPD-L1 in cynomolgus may thus be caused by CCX559 enhancing ongoing inflammation in one or more tissues, leading to upregulation of PD-L1 expression, shedding, or exosomes.

The preclinical pharmacology studies support the examination of several peripheral biomarkers for PD-L1 engagement and activity, including CD4^+^ and CD8 T cell proliferation, as well as IFNγ, sPD-L1, and other cytokines and chemokines. PD-L1 cellular localization within biopsies pre-and post-treatment may provide evidence of CCX559 target engagement within patient tumors.

In conclusion, we believe that our *in vitro* and *in vivo* studies support the therapeutic use of CCX559 as a PD-L1 inhibitor in patients with solid tumors. We further anticipate that the unique mechanism and small molecule properties of CCX559 may increase the pool of patients that respond to PD-L1 therapy. CCX559 is currently in clinical development with a Phase 1, first in patient, multicenter, open-label, dose-escalation study in subjects with solid tumors (ACTRN12621001342808).

## Materials and methods

Data points underlying the graphs in Figs 1–7 are fully available in S2 Appendix.

### Binding and cell based assays

Readouts for all *in vitro* assays were measured on a FlexStation3 (Molecular Devices) unless otherwise stated. Additional reagent sources are listed in the appendix (S1 Appendix). For PD-1 binding assays, 1 μg/ml of human PD-L1 (hPD-L1) or 3 μg/ml human PD-L2 in PBS was coated on 96 well Maxisorp plates. Serial dilutions of compound or anti-hPD-L1 antibody (clone NAT105, BioLegend 367402) were added, followed by 0.3 μg/ml biotin-hPD-1, streptavidin–HRP, and TMB substrate. For CD80 interaction, plates were coated with 2 μg/ml goat anti-mouse IgG2a antibody, followed by 10 μg/ml hCD80-mFc fusion protein and 1.25 μg/ml biotin-hPD-L1. For the TCR reporter assay (Promega J1252), CHO-K1 cells, engineered to express human PD-L1 and a cell surface TCR activator, were seeded in 96 well plates overnight. Jurkat cells, which express PD-1 and the T cell receptor (TCR)-inducible luciferase reporter gene NFAT-RE, were added in 100% FBS along with compounds. After 6 hours Bio-Glo Reagent was added to detect Jurkat reporter activity. The PathHunter^®^ dimerization assay employed U2OS PD-L1/PD-L1 cells (Eurofins DiscoverX, 93-1129C3) seeded in 96 well white bottom plates.

To measure PD-L1 internalization, MC38-hPD-L1 cells (GenOway S.A.) and RKO cells were seeded on cell culture slides or in 96 well flat-bottom plates. After incubation with compounds or antibodies, cells were fixed with 4% paraformaldehyde, followed by 0.3% Triton X-100 for total or buffer for cell surface-only staining. The cells were blocked with 5% normal goat serum and stained with 1 μg/ml anti-hPD-L1 monoclonal antibody 28–8 (Abcam, ab205921), 5 μg/ml DyLight 488-conjugated goat anti-rabbit secondary antibody and DAPI. Samples were kept in the dark and images were taken within 2 days on a Nikon Eclipse E800.

### Primary human immune cell assays

Human peripheral blood mononuclear cells (PBMCs) were isolated using a Ficoll gradient in StemCell SepMateTM-50 tubes from healthy volunteers using blood processed in Leukoreduction System (LRS) chambers (Stanford Blood Center, CA). Following red blood cell lysis (BD Biosciences, 555899), PBMCs were used immediately or stored in liquid nitrogen in 90% FBS/10% DMSO. Monocytes isolated with human CD14^+^ MicroBeads (Miltenyi, 130-045-201) were differentiated to dendritic cells (moDCs) with GM-CSF (100 ng/ml) and IL-4 (50 ng/ml) for 6 days. Maturation of moDCs was induced with IL-6 (2000 IU/ml), IL1β (400 IU/ml), TNFα (2000 IU/ml) and PGE2 (2 μg/ml).

For the mixed lymphocyte reaction, matured moDCs and CD4^+^ T cells from different human donors were cultured in a 1:10 cell ratio in DMEM/10% FBS for 5 days in 96-well flat-bottom cell culture plates, after which IFNγ secretion was measured (R&D Systems, DuoSet^®^ ELISA DY285B). Compounds were added to moDCs 30 minutes prior to the CD4+ T cells, which were freshly isolated with human CD4+ MicroBeads (Miltenyi, 130-050-101). For the PBMC-mediated tumor cell-killing assay, A375-eGFP cells (Imanis Life Sciences CL056-STAN) were seeded in 96-well clear-bottom black tissue culture-treated plates, followed by addition of compound or antibody. PBMCs, which had been stimulated with Staphylococcal Enterotoxin-B (SEB, EMD Millipore, 324798) for three days, were added to the wells (PBMC:A375-eGFP ratio 2:1) and incubated for 120 hours.

For the cytokine release assay, freshly isolated human PBMCs (hPBMCs) from 5 healthy donors were treated with compound alone or with CD2/CD3/CD28 beads (Miltenyi, 130-091-441). After 48 hours cytokine secretion was quantitated with a Luminex^®^ multiplex assay (R&D Systems, Catalog No. LXSAHM-19) on a MagPix^®^ xMAP^®^ Instrument. Concentrations were calculated using standards fit with a five-parameter curve in the Belysa^™^ analysis software.

### Murine *in vivo* studies

C57BL/6 wild type (Charles River Laboratories, Hollister, California), human PD-L1 knock-in (hPD-L1 KI, GemPharmatech) mice, and NOD.CB17-PrkdcScid/NCrHsd (NOD.SCID, Envigo, Livermore, CA) mice were housed at the ChemoCentryx animal facility at least 1 week prior to study start, in accordance with the Guide for the Care and Use of Laboratory Animals of the National Research Council. All study plans were prepared in advance under a protocol approved by the ChemoCentryx Institutional Animal Care and Use Committee, and recorded with Study Director software (v3.1.399, Studylog Systems Inc). For all studies the experimental unit was a single animal, and the negative control groups were vehicle only for CCX559 and the matching isotype human IgG1κ antibody (CrownBio, C0001-3) for MEDI4736 (CrownBio, custom made). Once dosing was initiated, all live mice were included in data measurement and analysis.

For MC38-hPD-L1 tumor studies, 5x10^5^ cells were injected subcutaneously into the right flank of 8-week-old wild type or hu-PD-L1 KI female mice on day 1. The mice were monitored daily for morbidity and mortality, including changes in body weight. The outcome measure was tumor volume, calculated as (L x W x W) / 2, where L is the longest tumor dimension and W the longest dimension perpendicular to L as measured with a digital caliper. On the day prior to dosing initiation, mice bearing tumors within the pre-designated size range were enrolled in the treatment groups (n = 10) based on random assignment with Matched Distribution randomization in Study Director to ensure uniform distribution of tumor sizes across groups. CCX559 or vehicle alone were dosed by oral gavage once daily, and CCX559 concentration was measured in plasma at day 3 of dosing and end of study using mass-spectrum analysis. MEDI4736 and the isotype control antibody were dosed by intraperitoneal injection every 5 days for a total of three doses in the wild type mice.

The A375 xenograft model procedure was similar to the MC38-hPD-L1 study, with the following differences. A 1:10 mixture of human PBMCs and A375 cells (9x10^5^ PMBCs: 9x10^6^ cells A375 cells) was injected subcutaneously into the right flank of 7-week-old NOD.SCID female mice on day 1. No mice were excluded from the study once inoculated. Dosing for all groups started on day 2, and antibody was given every 5 days for a total of 5 antibody doses per mouse.

For PD-L1 cell surface assessment, cells were isolated by finely chopping and meshing tumors through 200 μm and 70 μm sieves, then stained with Alexa Fluor 488 anti-mouse CD45 and phycoerythrin (PE) anti-hPD-L1 clone MIH1 (Thermo Scientific, 12-5983-42) and analyzed in a BD LSRFortessa^™^ cytometer. FACSDiva software was used to calculate the median fluorescent intensity (MFI) of surface PD-L1 on CD45-negative cells, after subtracting the background MFI on unstained cells. For immunohistochemistry, sections from formalin-fixed and paraffin embedded tumor were stained with anti-hPD-L1 clone 28–8 (1 μg/mL) on a Bond Rxm using the Bond Refine Detection kit, then imaged on a Nikon Ti2 microscope.

### Cynomolgus *in vivo* pharmacodynamics

A toxicology study in compliance with the Food and Drug Administration (FDA) Good Laboratory Practice (GLP) regulations for Nonclinical Laboratory Studies was performed at an Altasciences Preclinical Seattle LLC testing facility accredited by the Association for Assessment and Accreditation of Laboratory Animal Care (AAALAC). The facility had an Animal Welfare Assurance approved by the Office of Laboratory Animal Welfare (OLAW), was registered with the United States Department of Agriculture (USDA), and study oversight was provided by an Institutional Animal Care and Use Committee (IACUC). Cynomolgus monkeys were used to evaluate safety as the only non-human species that cross-reacts to CCX559: to that end, 36 non-tumor bearing animals (18 male, 18 female) were dosed in the study in order to meet the objective of adequately assessing safety prior to human clinical trials in accordance with guidelines from the International Conference on Harmonization (ICH) M3(R2), S3a, S9, and CPMP.

The animals were socially-housed in cages that comply with the Animal Welfare Act and recommendations set forth in the Guide for the Care and Use of Laboratory Animals (National Research Council 2011). Animals were randomly assigned to groups based on established social unit. Fresh drinking water was provided ad libitum, and fruits, vegetables, treats, as well as enrichment devices were provided throughout the course of the study.

A mortality check was conducted twice daily during the study to assess general animal health and wellness, along with a visual inspection for overall appetite. Cage side clinical observations were performed once daily, except on the one day per week when a detailed examination was performed for all animals. At the end of study, animals were sedated and euthanized by an overdose of euthanasia solution.

Enrolled cynomolgus monkeys were dosed once daily via nasogastric administration with CCX559 or control for 4 weeks. Plasma samples from the CCX559 25 mg/kg, 60 mg/kg, and vehicle dosed animal groups (n = 5 per gender) were analyzed for soluble PD-L1 on days 1 and 28 by ELISA (R&D Systems, FCSTM21-13).

## Supporting information

S1 Fig CCX559 was selective for human PD-L1 in *in vitro* binding and cell based assays. (DOCX)

S2 Fig CCX559 target engagement on the MC38-hPD-L1 tumor cell surface. (DOCX)

S3 Fig CCX559 post-dosing study MC38-hPD-L1 tumor volumes and PD-L1 detection. (DOCX)

S4 Fig CCX559 did not induce the release of IFNγ or IL-6 from human PBMCs in the absence of T cell activation. (DOCX)

S5 Fig CCX559 cross-reacted with cynomolgus PD-L1 and increased IL-6 plasma levels in cynomolgus monkeys. (DOCX)

S1 Appendix Materials and methods. (DOCX)

S2 Appendix Data corresponding to Figs 1–7 graphs. (XLSX)
