# Supplementary material for: CCX559 is a potent, orally-administered small molecule PD-L1 inhibitor that induces anti-tumor immunity
Source: PLoS One. 2023 Jun 7;18(6):e0286724. doi: 10.1371/journal.pone.0286724 (PMC10246841; doi:10.1371/journal.pone.0286724)
Supplement: S1 Fig — (DOCX) [file pone.0286724.s001.docx]

**A**

**B**

**C**

**D**

**E**

**F**

**Fig S1. CCX559 was selective for human PD-L1 in *in vitro* binding and cell based assays.**

(A) Human PD-1 binding to immobilized human PD-L2 was inhibited by incubation with an anti-hPD-1 antibody. (B) CCX559 did not prevent human TIGIT binding to plate-bound CD155 (green circles). A human TIGIT antibody blocked the interaction of TIGIT with CD155 (blue squares), confirming the assay detected inhibitors. (C) Jurkat cells with the NFAT-reporter were co-cultured with 293 cells that expressed a TCR activator but not PD-L1. No effect of CCX559 (green circles) or an anti-PD-L1 antibody (blue squares) was observed on TCR signaling. (D) Mouse PD-1 binding to immobilized mouse PD-L1 was not affected by treatment with up to 1 μM CCX559. (E) A mouse PD-L1 antibody blocked mouse PD-1/PD-L1 binding as a positive control for the assay in D. (F) CCX559 inhibited soluble mouse PD-1 protein binding to immobilized human PD-L1, with an IC_50_ comparable to the interaction with human PD-1 (1.0 nM, 95% confidence interval 0.59 nM to 1.7 nM). IC_50_ values were calculated with GraphPad Prism using 3 parameter nonlinear regression.
