# Supplementary material for: CCX559 is a potent, orally-administered small molecule PD-L1 inhibitor that induces anti-tumor immunity
Source: PLoS One. 2023 Jun 7;18(6):e0286724. doi: 10.1371/journal.pone.0286724 (PMC10246841; doi:10.1371/journal.pone.0286724)
Supplement: S2 Fig — (DOCX) [file pone.0286724.s002.docx]

**A**

**B**

**C**

**Fig S2. CCX559 target engagement on the MC38-hPD-L1 tumor cell surface.**

(A and B) MC38-hPD-L1 cells were treated *in vitro* with CCX559 or the inactive control compound (A), MEDI4736 or an isotype-matched antibody (B) and then stained with PE-conjugated anti-hPD-L1 clone MIH1. Dose dependent competition of CCX559 and MEDI4736 with MIH1 was detected as a reduction in median fluorescent intensity (MFI) by using flow cytometry. IC_50_ values were calculated with GraphPad Prism using 3-parameter nonlinear regression. (C) In the MC38-hPD-L1 study, CCX559 concentration in blood plasma was measured on day 13, 3 days after dosing was initiated and 24 hours after the previous dose.
