# Supplementary material for: CCX559 is a potent, orally-administered small molecule PD-L1 inhibitor that induces anti-tumor immunity
Source: PLoS One. 2023 Jun 7;18(6):e0286724. doi: 10.1371/journal.pone.0286724 (PMC10246841; doi:10.1371/journal.pone.0286724)
Supplement: S3 Fig — (DOCX) [file pone.0286724.s003.docx]

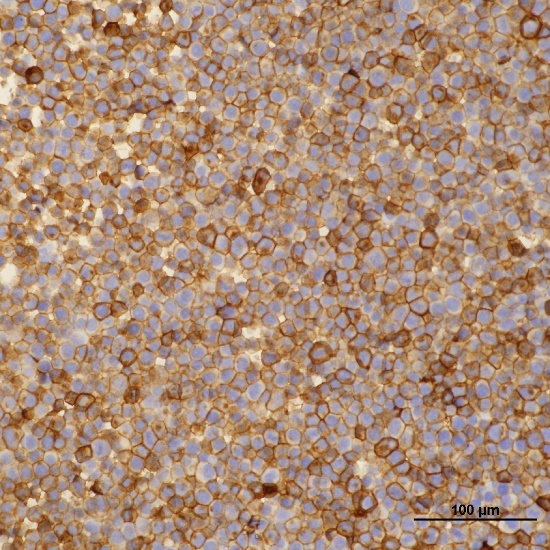

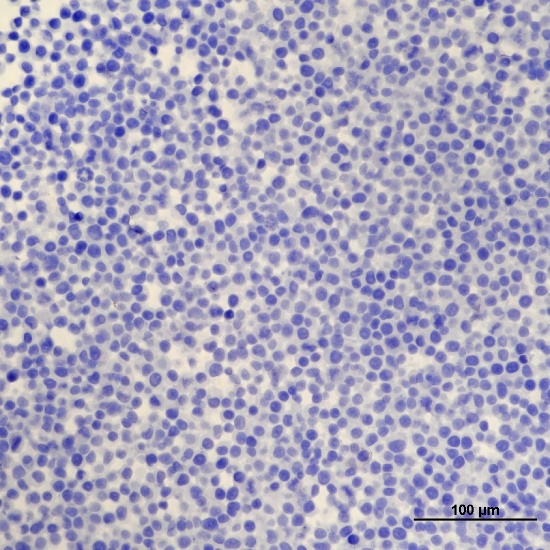


**A**

**C**

**B**

**Fig S3. CCX559 post-dosing study MC38-hPD-L1 tumor volumes and PD-L1 detection.**

(A) Tumor volume changes observed for the study shown in Figure 5B-H. The 7 day period of once per day CCX559 (purple circles, n=15) or vehicle (black squares, n=5) oral dosing is indicated by brackets. On day 23 after 7 days of CCX559 treatment, tumor volumes were significantly reduced compared to vehicle treatment (p = 0.005, Mann Whitney test). On days 1, 5, and 12 post dose (days 23, 28 and 35 post tumor cell inoculation), tumors were collected for IHC and drug level assays (n = 5 mice for CCX559, n = 2 mice for vehicle). (B and C) IHC validation for the anti-PD-L1 antibody clone 28-8 showed strong plasma membrane staining of FFPE MC38-hPD-L1 cell pellets (B) compared to isotype control (C).
