# Supplementary material for: CCX559 is a potent, orally-administered small molecule PD-L1 inhibitor that induces anti-tumor immunity
Source: PLoS One. 2023 Jun 7;18(6):e0286724. doi: 10.1371/journal.pone.0286724 (PMC10246841; doi:10.1371/journal.pone.0286724)
Supplement: S4 Fig — (DOCX) [file pone.0286724.s004.docx]

**A**

**B**

**Fig S4. CCX559 did not induce the release of IFNγ or IL-6 from human PBMCs in the absence of T cell activation.**

(A and B) The effect of CCX559 at 80 nM, 0.4 μM and 2 μM, alone or in the presence of anti-CD3/CD28/CD3 microbeads, was examined in a cytokine release assay with PBMCs from five donors. All donor PBMCs secreted both IFNγ (A) and IL-6 (B) when stimulated by the CD3/CD28/CD3 beads for 48 hours, but CCX559 treatment alone induced no cytokine secretion.
