# Supplementary material for: CCX559 is a potent, orally-administered small molecule PD-L1 inhibitor that induces anti-tumor immunity
Source: PLoS One. 2023 Jun 7;18(6):e0286724. doi: 10.1371/journal.pone.0286724 (PMC10246841; doi:10.1371/journal.pone.0286724)
Supplement: S5 Fig — (DOCX) [file pone.0286724.s005.docx]

**A**

**B**

**C**

**E**

**D**

**Figure S5: CCX559 cross reacted with cynomolgus PD-L1 and increased IL-6 plasma levels in cynomolgus monkeys.**

(A) CCX559 inhibited immobilized cynomolgus PD-L1 binding to soluble cynomolgus PD-1 *in vitro*. (B and C) IFNγ levels were increased in the supernatant of SEB-exhausted cynomolgus monkey PBMCs treated with CCX559 (B, green circles) compared to an inactive control compound (B, black circles), and MEDI4736 (C, blue squares) compared to an isotype control antibody (C, grey squares). (D and E) The change in plasma IL-6 levels from baseline were calculated for each individual male (D) or female (E) animals (n=5 per gender). The time points include: C_max_ (4 hours post dose) and trough (24 hours post dose) on day 1 and day 28 for all animals of both genders, and on day 48, 20 days after the last dose, for 2 animals per gender. The average predose level of IL-6 across groups ranged from 2.5 to 7 pg/ml.
