# Supplementary material for: CCX559 is a potent, orally-administered small molecule PD-L1 inhibitor that induces anti-tumor immunity
Source: PLoS One. 2023 Jun 7;18(6):e0286724. doi: 10.1371/journal.pone.0286724 (PMC10246841; doi:10.1371/journal.pone.0286724)
Supplement: S1 Appendix — (DOCX) [file pone.0286724.s006.docx]

**Appendix: Materials and Methods**

**Reagents and consumables**

Antibody and protein sources: human PD-L1 (R&D systems, 156-B7); biotinylated human PD-1 and PD-L2 protein (ACRO Biosystems, PD1H82F2 and PD2-H5251); human CD80 and PD-1 ectodomains fused to mouse Fc (Chimerigen Laboratories, CHI-HF-211CD80 and CHI-HF-211PD1); MIH1 (Phycoerythrin (PE)-conjugated, Thermo Scientific, 12-5983-42); goat anti-mouse IgG2a antibody (SouthernBiotech, 1081-01); DyLight 488-conjugated goat anti-rabbit secondary antibody (Vector labs, DI-1488). Cytokines were from Peprotech (catalog numbers): GM-CSF (300-03); IL-4 (200-04); IL-6 (200-06); IL-1β (200-01B); TNFα (300-01A); PGE2 (P0409). CCX559 (lots 5E and 14A) was prepared by the Medicinal Chemistry Department at ChemoCentryx and stored as powder or a 10 mM solution in dimethyl sulfoxide (DMSO) at -20 °C.

Consumables and reagents for plate-based binding assays: 96 well Maxisorp plates (Thermo Scientific, 439454); 96 well white bottom TC-treated plates (Costar, 3917); 96 well flat-bottom cell culture plates (Thermo Scientific, 163320); PBS (Corning, 21-031-CM); 2% BSA (SIGMA, A7979); TWEEN-20 (Sigma, P1379); Triton X-100 (Sigma, 93443); streptavidin–HRP (Corning, 21-031-CM); TMB substrate (BD OptEIA™, BD Biosciences, 555214); 5% normal goat serum (Jackson ImmunoResearch, 005-000-121); 4% paraformaldehyde (Thermo Scientific, J19943-K2); Prolong Gold Antifade mounting medium with DAPI (Invitrogen, P36935); DAPI (Invitrogen, D1306). Cynomolgus plasma IL-6 was measured with non-Human Primate XL Cytokine Luminex® assays (R&D Systems, DB7H10 and FCSTM21-13).

**Plate based binding assays**

Protocols for the following assays were similar to PD-1/PD-L1, with the changes noted below.

Human TIGIT/CD155: Assay plates were coated with 5 μg/mL human CD155 (R&D systems, 9174-CD) and after compound or antibody treatment 0.3 μg/mL Biotin-hTIGIT (ACRO Biosystems, TIT-H82F1) was added. An anti-hTIGIT antibody (R&D systems, MAB7898) was used to validate the assay.

Mouse or human PD-L1/mouse PD-1: Plates were coated with 1 µg/mL of recombinant human or mouse PD-L1 (ACRO, PD1-M5251), and after incubation with compound 1 µg/ml biotinylated mouse PD-1 protein (ACRO, PD1-M8259) was added.

Cynomolgus PD-L1/PD-1: Plates were coated with 1 µg/ml cynomolgus monkey PD-L1 protein (ACRO, PD1-C5253), and 1 µg/ml biotinylated cynomolgus monkey PD-1 (Sino Biological, 90311-C41H-B) was added after compound or antibody incubation.

**Cell Culture**

CHO-K1 cells expressing human PD-L1 and TCR activator were cultured in Ham’s F-12 medium supplemented with 10% FBS and 1x Pen-Strep (penicillin-streptomycin antibiotic solution provided as a 100-fold concentrate). Jurkat cells were grown in RPMI/10% FBS/Pen-Strep. The aAPC/CHO-K1 and Jurkat transgenes were maintained with Hygromycin B (200 µg/ml) and G418 (500 µg/ml), respectively. A375-eGFP cells, MC38-hPD-L1 cells, and RKO cells (ATCC, CRL-2577) were cultured in DMEM with 10% FBS/Pen-Strep. For A375-eGFP cells 1 μg/ml of puromycin was added for maintaining eGFP transgene. Primary immune cells were cultured in RPMI-1640 (Corning, 10-041-CM) with 10% FBS/Pen-Strep unless otherwise stated. For the NFAT reporter cell assay, control cells were prepared as follows: 293 cells (ATCC, Catalog No. CRL-1753) were cultured in DMEM/10% FBS/Pen-Strep and transfected with expression vector containing a TCR activator (BPS Bioscience, 79778) using lipofectamine (Invitrogen, 11668-027) when the cells were 90% confluent.

**Anti-PD-L1 clone MIH1 competition with CCX559**

MC38-hPD-L1 cells were detached with 0.25% trypsin and resuspended in culture medium at a concentration of 1x10^6^ cells/ml, then 200 μl cells were put in each well of a 96-well assay plate. CCX559,  the inactive control compound, MEDI4736 or an isotype-matched control antibody were added to the wells and incubated on ice for 2 hours. Aliquots of 50 μl cells from each well were stained with a PE-conjugated anti-hPD-L1 antibody clone MIH1 or PE-conjugated isotype-matched control antibody diluted 1:200 for 30 minutes on ice. Cells were then washed twice with PBS/10% FBS/0.1% sodium azide and analyzed on a BD LSRFortessa™ cytometer. Mean fluorescence intensity (MFI) was calculated using FlowJo software (FlowJo LLC). IC_50_ values were calculated with GraphPad Prism using 3 parameter nonlinear regression.

**Murine *in vivo* studies**

To ensure consistent dosing of CCX559, drug levels were measured in circulation by collecting blood samples after three days of dosing and at the end of the study, 24 hours after the previous dose. A single operator was responsible for assigning animals according to the randomization results, and for measurement of tumor volume.

**Cynomolgus SEB T cell assay**

Male cynomolgus monkey PBMCs were isolated from whole blood (BioIVT) by density gradient centrifugation. 50 ml of blood with ACD-A from each donor monkey was split into 3 aliquots, diluted with PBS to a final volume of 35 ml, and carefully layered over 14 ml Ficoll-Paque Plus in the StemCell SepMateTM-50 tube. From this point onward the blood was processed similarly to human PBMCs. The cyno PBMCs were resuspended at 2x10^6^ cells/ml in RPMI-1640/10% FBS/Pen-Strep, seeded at 200 μl in 96-well round bottom tissue-culture plate wells, then treated with 100 ng/ml of Staphylococcal Enterotoxin B (SEB) for three days.  Compounds or antibodies were added with fresh SEB (0.1 ng/mL) to the relevant wells. After 5 days of incubation at 37 °C in 5% CO_2_, IFNγ was measured in cell culture medium using a primate IFNγ DuoSet^®^ ELISA kit (R&D Systems, DY961) according to manufacturer’s specifications.
